# Bending properties of human cartilaginous ribs and costal cartilage material vary with age, sex, and calcification

**DOI:** 10.1093/jbmrpl/ziae153

**Published:** 2024-12-03

**Authors:** Megan H Goh, Dennis E Anderson

**Affiliations:** Center for Advanced Orthopaedic Studies, Beth Israel Deaconess Medical Center, Boston, MA, 02215, United States; Department of Orthopaedic Surgery, Harvard Medical School, Boston, MA, 02115, United States; Center for Advanced Orthopaedic Studies, Beth Israel Deaconess Medical Center, Boston, MA, 02215, United States; Department of Orthopaedic Surgery, Harvard Medical School, Boston, MA, 02115, United States

**Keywords:** cadaveric tissue, mechanical testing, soft tissue biomechanics, pQCT, aging

## Abstract

Costal cartilage plays an important functional role in the rib cage, but its mechanical properties have not been well characterized. The objective of this study is to characterize the properties of human costal cartilage and examine the effects of age, sex, rib level, and degree of calcification. We obtained cadaveric costal cartilage samples of ribs 3-6 with intact perichondrium from 24 donors (12 females and 12 males) evenly distributed by age (range 47-94 yr). Peripheral QCT scans were used to quantify geometric properties (area moments) and tissue calcification (as volume, length, and classified as central, peripheral, and mixed). Four-point bending tests were performed on each sample, and bending stiffness and modulus outcomes were evaluated by fitting data from mechanical testing with non-linear pseudo-elastic models (composed of linear and cubic components, separated into loading and unloading regimes). Effects of sex, age, rib level, and cartilage calcification on bending stiffness and modulus outcomes were assessed with mixed-effects regression models. Cartilage size (area moment) was larger in males than females and positively associated with age, while there was more calcification volume in cartilage of females than males. During loading, stiffness (linear and cubic) was larger in males, while modulus (linear and cubic) was larger in females. Linear stiffness and modulus were both negatively associated with age, positively associated with calcification, and varied between rib levels. Cubic (nonlinear) components of stiffness and modulus were positively associated with calcification and varied by rib, while modulus (but not stiffness) was negatively associated with age. During unloading, the linear stiffness and modulus values were much lower, though some similar associations were found. Overall, this study adds to our understanding of the behavior of costal cartilage as a nonlinear visco-elastic material, and the effects of sex, aging, and calcification on mechanical behavior.

## Introduction

Costal cartilage plays an important structural role in the thorax as it connects the ribs to the sternum. It is composed of hyaline cartilage, a form of connective tissue made up of proteoglycans, collagen fibrils, and chondrocytes,^1^ surrounded by perichondrium, a layer of dense fibrous tissue lined with cells that support the maintenance of the hyaline matrix.^2^^,^^3^ Clinically, costal cartilage has been used as a source of grafting material for auricular,^4^^,^^5^ nasal,^6^^,^^7^ and laryngotracheal reconstructive procedures.^8^ Due to its durable and flexible composition, costal cartilage reinforces the structural integrity of the ribcage through its elastic and deformable nature,^9^ facilitates the dynamic mechanics of respiration,^10^ and provides protection for internal organs.^11^ Costal cartilage occurs anatomically as cartilaginous ribs, extensions of the bony ribs, without which the rib cage would not be fully intact. Thus, it is not surprising that the costal cartilage contributes significantly to the stiffness of the thoracic structure.^12^ However, despite the numerous physiologic and clinical purposes of costal cartilage, there remains comparatively little study of its mechanical properties. Better characterization of the biomechanical properties of costal cartilage would be beneficial for understanding aging-related or disease-related functional changes in the thorax, assessments of injury risk, and even to improve treatments, such as the design of biomimetic thoracic implants that are often used in reconstructive surgeries following extensive tumor resection or trauma.^9^^,^^13^

Multiple studies have reported on the mechanical or material properties of costal cartilage in some form. However, results are variable, likely due to variations in testing methods, tissue samples, and non-linear tissue responses. The various studies reporting on the tissue-level biomechanical properties of costal cartilage^14–19^ have used indentation, tensile, compression, and bending tests. Most of these tests involve the biomechanical characterization of small, excised pieces of costal cartilage, but preparation methods vary. A wide range of resulting values for tissue elastic modulus has been reported, from 0.4 to 66 MPa.^9^^,^^20^ Finite element studies of the rib cage have utilized a wide range of elastic modulus values for costal cartilage, ranging from 24.5 to 480 MPa.^21^^,^^23^ Most studies evaluating costal cartilage properties have not explicitly considered nonlinear or viscoelastic material behavior in their analyses, which may also help explain variations in reported material properties. Moreover, while the material properties of costal cartilage tissue have been reported from several studies, the bending properties of intact cartilage specimens have not been widely reported.

Several studies have explored the role of age-related changes on the biomechanical properties of costal cartilage.^17–19^ It may be supposed that aging would increase cartilage stiffness, as older adults exhibit nearly three times less thoracic motion in vivo than young adults, indicative of increased thoracic stiffness with aging.^24^ While the causes of increased thoracic stiffness in older adults are surely multi-factorial, older adults exhibit more calcification within the costal cartilage^25^ and osteophytes in the sternocostal joints,^26^ which may stiffen these components of the thorax. Indeed, calcification of costal cartilage could impact its mechanical properties.^27^^,^^28^ Through aging, costal cartilage also undergoes “amianthoid transformation,” where there is an increase in the diameter of the collagen fibers within the hyaline cartilage.^29^ However, studies of the effects of age and degree of calcification on costal cartilage stiffness have produced variable results. Lau et al.^17^ found no effect of age or calcification on costal cartilage stiffness through indentation testing. Conversely, through indentation and unconstrained compression, Weber et al.^19^ found that there was a decrease in the Young’s modulus of costal cartilage with increasing age. However, studies of costal cartilage properties to date have not sought a well-composed sampling of specimens by age, and an analysis of costal cartilage properties accounting for both age and calcification across a wide range of ages is lacking.

The aims of this study were to: (1) determine geometric properties (cross-sectional area and area moments), density, and calcification of human cadaveric costal cartilage specimens and their associations with age and sex; and (2) determine the bending stiffness and bending (material) modulus of human cadaveric costal cartilage specimens, and whether these properties varied with age, sex, rib location, and costal cartilage calcification. We hypothesized that male sex would be associated with larger geometric properties and age with increased density and calcification. Further, we hypothesized that male sex, age, and larger amounts of calcification would be associated with higher measured bending stiffness of whole costal cartilage samples, while age and calcification would be associated with larger bending modulus.

## Materials and methods

### Specimen selection and power analysis

Preliminary power analysis was based on in vivo data^24^ suggesting a strong association of age on thoracic stiffness (which translates to an *R*^2^ of about 0.35). Assuming an effect size of *R*^2^ = 0.35, a multiple regression analysis with two independent variables (age and calcification) would require a sample size of 21 to achieve 80% power. Thus, we planned to obtain rib cage specimens from 24 adult human cadavers. Assuming four costal cartilage samples per specimen, we would be able to detect significant results with *R*^2^ as low as 0.1 with 80% power. In order to provide a wide and relatively even distribution of specimens by age and sex, specimens were sought from six tissue donors within each of four age ranges (40-55; 56-70; 71-85; 86-100), with three male and three female within each age group.

**Figure 1:**
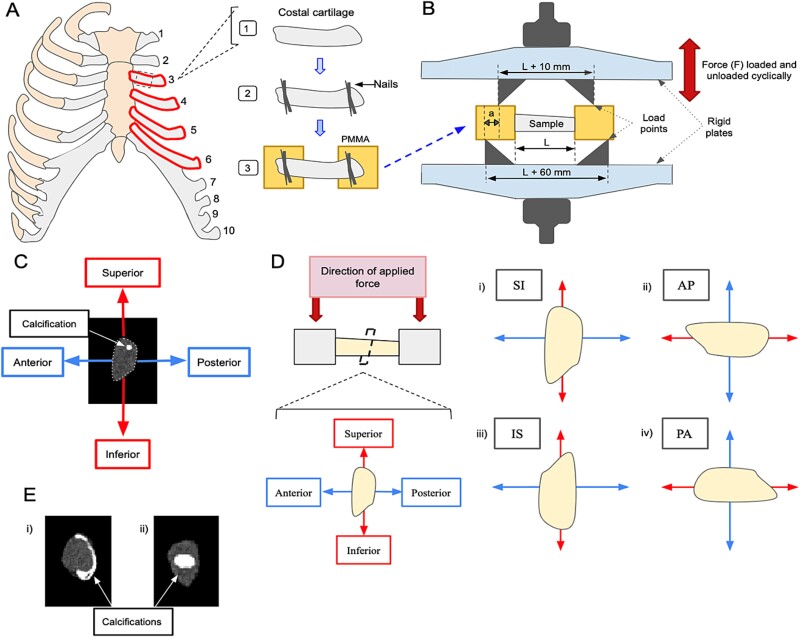
(A) Schematic of sample preparation procedure. Costal cartilage was excised from ribs 3-6. Small nails were placed at either end of the sample before being embedded in polymethylmethacrylate (PMMA) cement. (B) Experimental setup for four-point bending test. The PMMA potted ends of the costal cartilage samples were placed in the four-point bending apparatus. Force (*F*) was cyclically loaded and unloaded for three cycles in displacement control. *L* is the length of the specimen and *a* is the distance between the upper and lower loading points. (C) Example of a cross-sectional slice of a costal cartilage specimen from a pQCT scan, showing areas of calcification. The area moment properties of the specimen were calculated about the anterior–posterior (AP) and superior–inferior (SI) axes indicated. (D) Cross-section of costal cartilage showing the orientation of the sample within the four-point bending apparatus. The force was applied downward during the bending test. Each sample was rotated 90° such that a different aspect (superior (i), anterior (ii), inferior (iii), and posterior (iv)) of the costal cartilage was facing upwards in the apparatus. (E) Example of costal cartilage calcification patterns: peripheral (i) and central (ii).

Fresh frozen cadaveric human rib cages of 24 donors (12 males and 12 females) with characteristics as described in Table 1 were obtained from a tissue bank (Anatomy Gifts Registry). Reported causes of death were primarily due to cancer (*n* = 13) and cardiovascular problems (*n* = 5), with the remainder including ALS (*n* = 1), GI bleeds (*n* = 1), and other or non-specified reasons (*n* = 4). The costal cartilage specimens of the third to sixth ribs on the left side were dissected from all the rib cages and cleaned. The specimens were then potted at both ends in polymethylmethacrylate (PMMA) cement, with a span of costal cartilage between the two pots (Figure 1). Before potting, two small nails were penetrated through the cartilage at each end to help reduce motion between cartilage and potting material during testing. Specimens were stored frozen, wrapped in 0.9% saline-soaked gauze, and thawed prior to testing and sprayed regularly during testing. The specimen length, defined as the distance of the span between the pots, was measured with calipers and recorded.

**Table 1:** Donor characteristics (mean + SD [minimum, maximum]) of cadaveric human rib cage specimens, by sex.

Sex	Age (yr)	Height (m)	Weight (kg)	BMI (kg/m^2^)
**Female (n = 12)**	71 ± 15 [48, 94]	1.62 ± 0.06 [1.52, 1.68]	67.2 ± 19.4 [40.8, 104.3]	25.7 ± 7.8 [15.9, 39.1]
**Male (n = 12)**	71 ± 17 [47, 93]	1.76 ± 0.09 [1.57, 1.85]	68.0 ± 14.9 [49.9, 99.8]	22.0 ± 5.3 [14.5, 35.5]

### Peripheral QCT (pQCT) scans and calcification assessment

All specimens were scanned cross-sectionally in a pQCT scanner (STRATEC XCT Research SA+) along the exposed length of the specimen with 0.5 mm in-plane and 2 mm slice resolution to quantify specimen geometric properties and amount of calcification. The cartilage was segmented in the scans using a custom program in MATLAB (The Mathworks, Inc.). Scan segmentation data was used to calculate second and fourth moments of area about the superior–inferior and anterior–posterior axes. Moments of area were calculated at each slice of the volumetric pQCT scan, and the equivalent area moment for the entire specimen length was estimated as the harmonic mean across all the slices. The coefficient of variation of area moment (CVAM) across all the slices was also determined as an estimate of the uniformity of the cross-section. Following Lau et al.,^27^ calcification was segmented from cartilage material using a density threshold of 285 mgHA/cm^3^. The amount of calcification for a specimen was quantified by the volume of calcification as a percentage of total specimen volume. The calcification morphology was further assessed in terms of the length of the longest contiguous calcified structure as a percentage of total specimen length and by the pattern of calcification deposits, as central, peripheral, or mixed.^30^^,^^32^ The pQCT scans for each costal cartilage sample were individually reviewed by each author to classify patterns, and discrepancies were jointly re-reviewed for consensus. A peripheral pattern was defined as any calcification occurring primarily at the cross-sectional periphery of the sample, often appearing to form a partial “shell” (Figure 1E). Conversely, a central pattern was defined as calcification that was fully encapsulated by visible costal cartilage. A mixed pattern was determined in cases in which there were characteristics of both peripheral and central calcifications.

### Four-point bending tests

After scanning, the specimens underwent mechanical testing on a servo-hydraulic materials testing system (Instron 8511, Instron) using a four-point bending configuration applied to the pots (Figure 1B). The lengths between the fixtures in the testing device were set based on the measured length of the specimen. The upper fixture had a separation of the length plus 10 mm, while the lower fixture had a separation of the length plus 60 mm. The specimens were tested four times each, with a different side (anterior, posterior, superior, and inferior) facing upwards each time in a randomized order (Figure 1D). There were several minutes between directional tests for sample repositioning, which would also allow for relaxation recovery between tests. The cyclic test performed on the specimens included an initial calibration loading to 10 N, followed by three loading and unloading cycles that were displacement-limited. The maximum displacement limit was set as 0.109 × specimen length, corresponding to a cartilage curvature of approximately 0.5°/mm length. Testing was performed at a displacement rate of 0.1 mm/s, and data was collected at 100 Hz.

### Data processing

Force-displacement data measured by the Instron was transformed into moment-angle data. Applied moment *M*, assumed to be a pure moment applied to the specimen, was estimated as:


(1) \begin{equation*} M=\frac{F\times a}{2} \end{equation*}

where *F* is measured force and *a* (= 25 mm) is the horizontal distance between the upper and lower load points applied to each pot.

The total bending angle applied to the cartilage specimen was estimated as:


(2) \begin{equation*} \theta =2\;{\tan}^{-1}\left(\frac{v}{a}\right) \end{equation*}

where *v* is measured crosshead displacement from the beginning of the test.

The test was assumed to approximate the bending of a beam under pure moments applied at each end. To relate angle to moment, a non-linear viscoelastic material was assumed. For non-linear elastic response, a cubic stress–strain model was adopted:


(3) \begin{equation*} {\sigma}^e={E}_1\varepsilon +{E}_3{\varepsilon}^3 \end{equation*}

where *E*_1_ and *E*_3_ are linear and cubic elastic moduli. This form can be taken as a polynomial expansion of well-known exponential non-linear material models for soft tissues^33^^,^^34^ and both exponential and cubic functions have also been applied to fit passive behavior of joints.^35^^,^^36^

From beam bending theory, bending moment is related to axial stress, and thus strain, by:


(4) \begin{equation*} M=-{\int}^y\left(\sigma dA\right)=-{\int}^y\left({E}_1\varepsilon +{E}_3{\varepsilon}^3\right) dA \end{equation*}

where *y* is the distance above the neutral axis of the element *dA* of the cross-sectional area.

Kinematically, the axial strain based on the applied bending angle is:


(5) \begin{equation*} \varepsilon =-\frac{y\theta}{L} \end{equation*}

where *L* is the length of the specimen (the span between the pots). Applying this relation, bending moment is related to applied angle as:


(6) \begin{equation*} M={\int}^y\left({E}_1\frac{y\theta}{L}+{E}_3\frac{y^3{\theta}^3}{L^3}\right) dA=\frac{E_1\theta }{L}{\int}^{y^2} dA+\frac{E_3{\theta}^3}{L^3}{\int}^{y^4} dA. \end{equation*}

Recognizing the portions in integrals as the second and fourth moments of area about the neutral axis, called *I*_2_ and *I*_4_, respectively, we are left with the following nonlinear model of applied moment:


(7) \begin{equation*} M\left(\theta \right)=\frac{E_1{I}_2}{L}\theta +\frac{E_3{I}_4}{L^3}{\theta}^3. \end{equation*}

Following Fung’s^37^ concept of pseudo-elasticity, which has been previously applied in costal cartilage modeling,^38^ we fit this non-linear elastic model separately to the loading and unloading portions of the testing. Thus, we are left with:


(8a) \begin{equation*} M\left(\theta \right)=\frac{E_{1L}{I}_2}{L}\theta +\frac{E_{3L}{I}_4}{L^3}{\theta}^3,\dot{\theta}>0 \end{equation*}

(8b) \begin{equation*} M\left(\theta \right)=\frac{E_{1U}{I}_2}{L}\theta +\frac{E_{3U}{I}_4}{L^3}{\theta}^3,\dot{\theta}<0 \end{equation*}

where the subscripts *L* and *U* denote moduli for loading and unloading behavior.

We define bending stiffness variables *K* such that eqn (8) can be written as:


(9a) \begin{equation*} M\left(\theta \right)=\frac{K_{1L}}{L}\theta +\frac{K_{3L}}{L^3}{\theta}^3,\dot{\theta}>0 \end{equation*}

(9b) \begin{equation*} M\left(\theta \right)=\frac{K_{1U}}{L}\theta +\frac{K_{3U}}{L^3}{\theta}^3,\dot{\theta}<0. \end{equation*}

**Figure 2:**
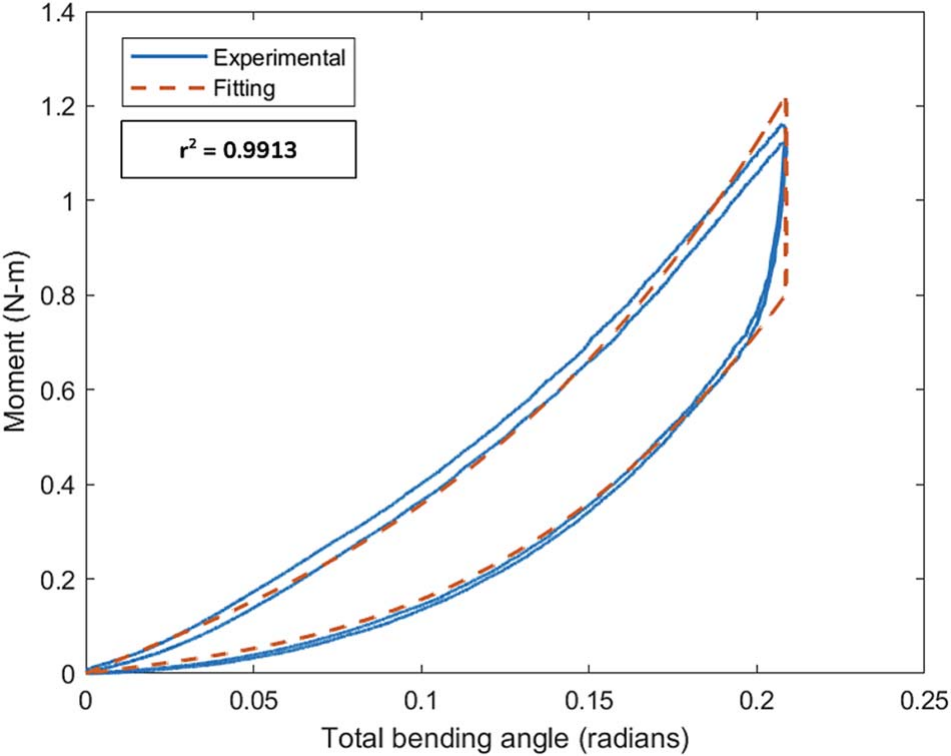
Example fitting of experimental data. The experimental data was acquired from the fifth rib with the anterior aspect of the costal cartilage orientated upwards in the 4-point bending apparatus. Fitting was performed on the second and third cycles of the test.

The testing measurements of force and displacement were zeroed to their values at the beginning of data collection and processed into moment and angular displacement data using eqns (1) and (2). In some tests, the crosshead lifted off the specimen during unloading cycles, and these data points were excluded when they were below a threshold of 0.1 N of measured force. Then, the models of eqn (9) were fit to the data, using custom scripts created in MATLAB (The Mathworks, Inc.). The second and third cycles of data from testing were used for fitting by default, unless there was a problem identified resulting in only one being used. The constants of eqn (9) were determined as the best fit in a least squares sense using a nonlinear constrained solver (fmincon), with the unloading stiffness constrained to be no greater than the loading stiffness (ie, *K*_1*U*_ ≤ *K*_1*L*_; *K*_3*U*_ ≤ *K*_3*L*_). Data and data fits were plotted for each test and reviewed to ensure that data collection, processing, and curve fitting had realized an acceptable fit (Figure 2). Specimen area moments determined by pQCT scans were then combined with the bending stiffness values to determine bending modulus values.

### Statistical analyses

For the first aim, the primary outcomes were the second moments of area of the specimen about the neutral axes, the amount of cartilage calcification as a percentage of specimen volume, the length of the calcification structure as a percent of specimen length, and the cartilage classifications. The second moments of area for each specimen were examined relative to neutral axes in the superior/inferior direction (*I*_2SI_) and in the anterior/posterior direction (*I*_2AP_)*.* The fourth moments of area (*I*_4SI_ and *I*_4AP_) were reported but not analyzed as separate outcomes (Table S1). Summary statistics were calculated at each rib level for each outcome. A multi-level mixed model regression was performed for each outcome variable with age, sex, and rib level as fixed effects, and donor as a random effect. Differences between ribs were examined with linear combinations of coefficients. For the second aim, the primary outcomes were bending stiffness (*K*_1*U*_, *K*_1*L*_, *K*_3*U*_, *K*_3*L*_) and bending modulus (*E*_1*U*_, *E*_1*L*_, *E*_3*U*_, *E*_3*L*_) values in loading and unloading. Based on univariate analyses, all three measures of cartilage calcification were weakly associated with stiffness and modulus outcomes (Table S2). Thus all were initially examined in multivariate analyses. Bending stiffnesses from complementary testing directions were combined for analysis—Anterior with Posterior, Superior with Inferior—to evaluate bending about their corresponding shared neutral axes. Bending moduli from all testing directions were combined for analysis to evaluate the underlying material properties of the costal cartilage. Summary statistics of each outcome were calculated by rib level and direction (for bending stiffness), separately in males and females. Multilevel mixed effects generalized linear models with a gamma distribution family and log linking function were fit for each outcome variable. Initially, age, sex, rib level, cartilage calcification volume, cartilage calcification length, and cartilage calcification classification were included as fixed effects, the CVAM as a covariate, and donor as a random effect. As only calcification volume was significant in any analysis, other measures of calcification were dropped from final analyses. Linear combinations of coefficients were used to examine differences between ribs and to obtain estimates of outcome variables for pure cartilage with no calcification and uniform cross section (ie, setting calcification percent volume and CVAM to 0). All analyses were performed in Stata/IC 13.1 (StataCorp, LP), with significance set at α = .05.

## Results

From a total of 96 costal cartilage specimens dissected (24 donors × 4 rib levels), 4 were not tested due to visible damage. An additional 10 specimens had one or more tests excluded from analysis due to lost data (*n* = 2), abnormal data or testing problems (*n* = 6), or damage observed to occur during an earlier test (*n* = 2). In one of these specimens, all four tests were excluded, as high measured loads were believed to indicate binding of the fixture during testing. Overall, data from 353 total tests performed on 91 specimens were included in the analyses.

### Area moment by axis, rib level, sex, and calcification

Specimen properties of length, area moments, and calcification are presented by rib level in Table 2. Area moments about both the AP axis (*I*_2AP_) and the SI axis (*I*_2SI_) were larger in males than females (*p* < .001). Area moment about the SI axis (*I*_2SI_) was larger in specimens with older age (*p* = .033), although the similar trend for *I*_2AP_ did not reach significance (*p* = .080). Between ribs, *I*_2AP_ was larger for rib 6 than rib 3 (*p* = .048), *I*_2SI_ was larger for rib 5 than rib 3 (*p* < .001) or rib 4 (*p* = .002), and *I*_2SI_ was larger for rib 6 than rib 3, rib 4, or rib 5 (all *p* < .001). Costal cartilage calcification by percent volume was higher in females than males (*p* = .042) and was higher in rib 4 than rib 6 (*p* = .002) but did not vary with age or between other ribs. No effects of age, sex, or level were found for calcification percent length.

**Table 2:** Specimen characteristics by rib level, separately for males and females.

Rib	Length (mm)	Area moment − AP (*I*_2AP_, mm^4^)	Area moment − SI (*I*_2SI_, mm^4^)	Calcification volume (%)	Calcification length (%)	Central/mixed/peripheral
**Male**						
**3**	26.3 ± 3.8	2855 ± 903	1016 ± 394	2.1 [0.2, 9.1]	52 [8, 100]	1/6/5
**4**	30.0 ± 6.2	3074 ± 960	1243 ± 493	2.4 [0.1, 16.7]	50 [7, 100]	3/4/5
**5**	29.8 ± 5.1	3082 ± 1046	1723 ± 597	2.3 [0.0, 15.7]	51 [0, 100]	1/4/6
**6**	38.9 ± 5.1	3268 ± 1292	2687 ± 933	3.7 [0.1, 8.5]	57 [10, 100]	1/5/6
**Female**						
**3**	22.6 ± 5.3	1417 ± 674	454 ± 250	7.3 [0.0, 34.5]	72 [0, 100]	1/6/3
**4**	25.9 ± 3.5	1409 ± 544	569 ± 233	7.4 [0.0, 37.6]	89 [0, 100]	2/6/3
**5**	27.1 ± 4.5	1389 ± 596	780 ± 458	10.0 [0.1, 23.8]	47 [8, 100]	3/7/2
**6**	35.9 ± 6.8	1500 ± 569	1220 ± 627	2.7 [0.0, 22.2]	55 [0, 100]	2/5/4

Calcification is presented as median [minimum, maximum], and other measures as mean ± SD. Calcification volume is percent of specimen volume that is calcified, and Length is the longest calcified structure as a percent of total specimen length. Central/mixed/peripheral shows the number of specimens per each classification; note that specimens with 0 calcification are not included in the classification.

### Bending stiffness

**Figure 3:**
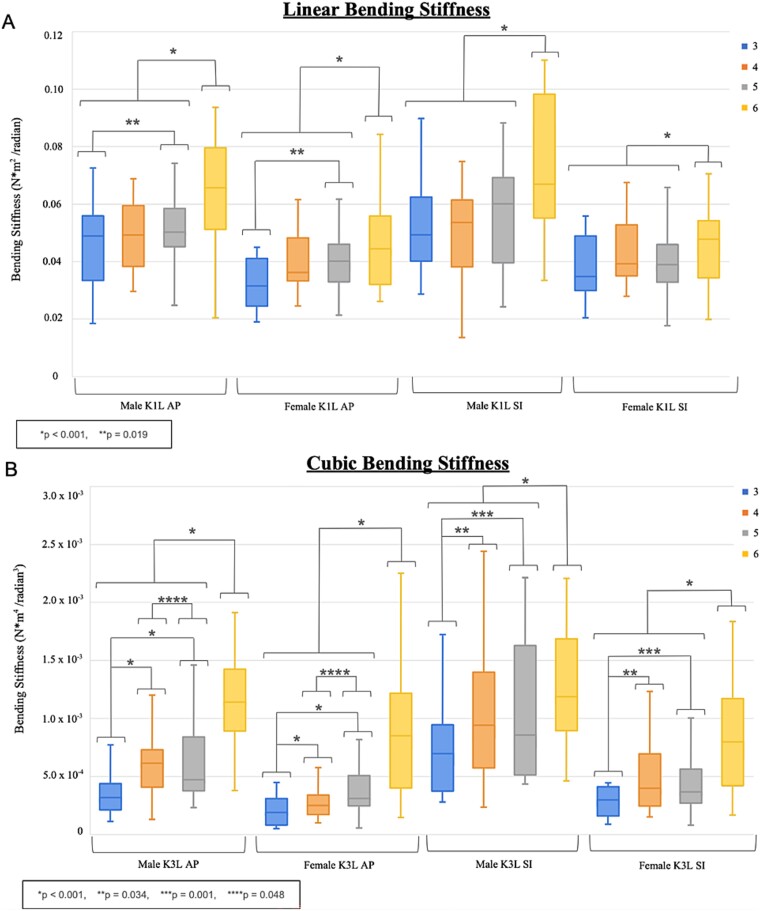
(A) Linear (K1L) bending stiffness in loading and (B) cubic (K3L) bending stiffness in loading averaged across rib levels along anterior–posterior (AP) and superior–inferior (SI) axes.

Specimen linear bending stiffnesses in loading (*K*_1*L*_) had a median [range] of 4.43 × 10^−2^ [1.84 × 10^−2^ to 1.16 × 10^−1^] N × m^2^/radian in the AP direction and 4.54 × 10^−2^ [1.36 × 10^−2^ to 1.10 × 10^−1^] N × m^2^/radian in the SI direction (Figure 3). It was larger in males than females for both AP and SI axes (*p* < .001), negatively associated with age for both the AP and SI axes (*p* < .001), and positively associated with amount of calcification for both the AP and SI axes (*p* < .001). Linear stiffnesses in loading of ribs 3, 4, and 5 were smaller than rib 6 about both AP and SI axes (*p* < .001 for all), and rib 3 was smaller than rib 5 about the AP axis (*p* = .019). CVAM was not a significant covariate for either AP or SI axes (*p* = .269 and *p* = .959, respectively).

Specimen cubic bending stiffness in loading (*K*_3L_) had a median [range] of 4.07 × 10^−4^ [4.65 × 10^−5^ to 2.25 × 10^−3^] N × m^4^/radian^3^ in the AP direction and 6.41 × 10^−4^ [8.36 × 10^−5^ to 4.19 × 10^−3^] N × m^4^/radian^3^ in the SI direction (Figure 3). It was larger in males than females for AP and SI axes (*p* < .001) and was positively associated with calcification through both the AP and SI axes (*p* < .001) but was not associated with age for either the AP or SI axis of applied force. Cubic stiffnesses in loading (*K_3L_*) of ribs 3, 4, and 5 were smaller than rib 6 about both AP and SI axes (*p* < .001 for all), rib 3 was smaller than rib 4 about both AP and SI axes (*p* < .001 and *p* = .034, respectively), rib 3 was smaller than rib 5 about both AP and SI axes (*p* < .001 and *p* = .001, respectively), and rib 4 was smaller than rib 5 about the AP axis (*p* = .048). CVAM was not a significant covariate for either AP or SI axes (*p* = .153 and *p* = .501, respectively).

Specimen linear bending stiffness in unloading (*K*_1*U*_) had a median [range] of 1.15 × 10^−2^ [3.28 × 10^−10^ to 5.80 × 10^−2^] N × m^2^/radian in the AP direction and 1.05 × 10^−2^ [1.60 × 10^−10^ to 6.36 × 10^−2^] N × m^2^/radian in the SI direction (Figure S1). It was negatively associated with age for both the AP (*p* = .001) and SI axes (*p* = .002), was not associated with calcification for either the AP or the SI directions (*p* = .452 and *p* = .879, respectively), and there was no sex difference for either AP or SI directions (*p* = .232 and *p* = .360, respectively). Linear stiffness in unloading (*K*_1*U*_) did not vary by rib level for either AP or SI axes (all *p* > .05). CVAM was not a significant covariate for either AP or SI axes (*p* = .718 and *p* = .271, respectively).

Specimen cubic bending stiffness in unloading (*K*_3*U*_) had a median [range] of 4.07 × 10^−4^ [4.65 × 10^−5^ to 1.88 × 10^−3^] N × m^4^/radian^3^ in the AP direction and 6.31 × 10^−4^ [8.36 × 10^−5^ to 3.12 × 10^−3^] N × m^4^/radian^3^ in the SI direction (Figure S1). It was larger in males than females for the AP and SI axes (*p* < .001) and positively correlated with calcification through both the AP and SI axes (*p* < .001), but not correlated with age in either axis of applied force. Cubic stiffness in unloading (*K*_3*U*_) of ribs 3, 4, and 5 was smaller than rib 6 about both AP and SI axes (*p* < .001 for all); rib 3 was smaller than ribs 4 and 5 about the AP axis (*p* < .001 for both) and SI axis (*p* = .026 and *p* = .001). CVAM was not a significant covariate for either AP or SI axes (*p* = .123 and *p* = .842, respectively).

### Bending moduli

**Figure 4:**
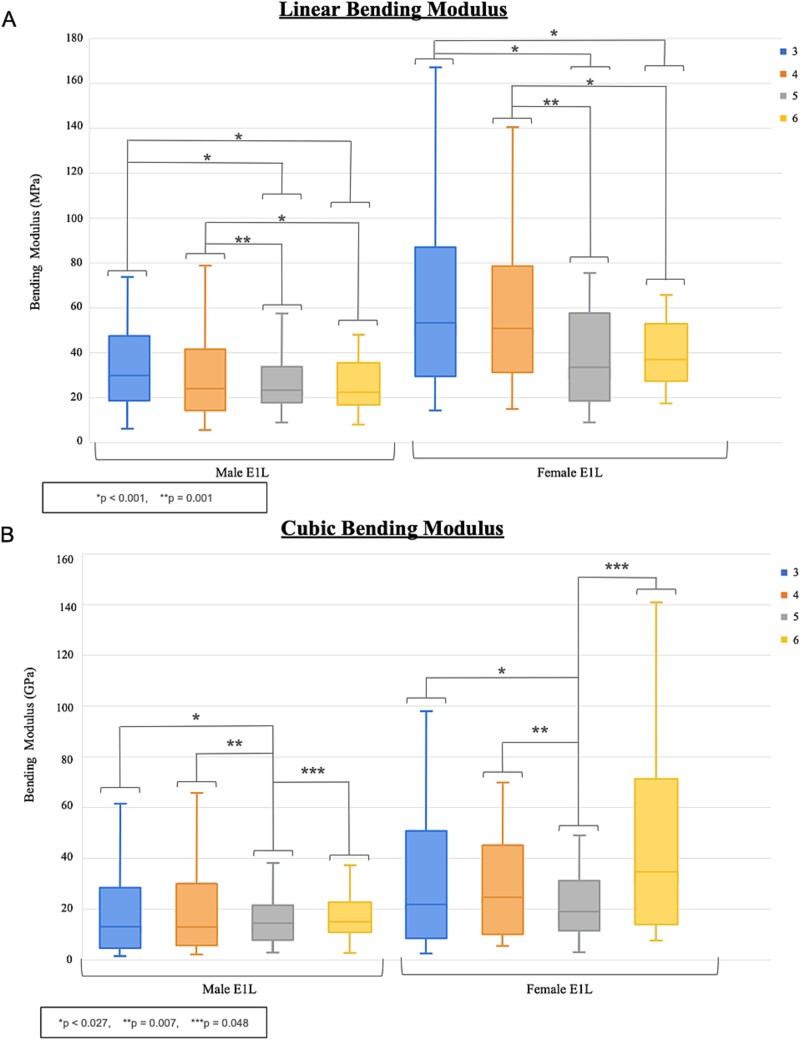
(A) Linear (E1L) bending elastic modulus in loading and (B) cubic (E3L) bending elastic modulus in loading by rib level and sex.

Specimen linear bending moduli in loading (*E*_1*L*_) had a median [range] of 34.0 [5.32-348] MPa (Figure 4). It was larger in females than males (*p* < .001), negatively associated with age (*p* < .001), and positively correlated with amount of calcification (*p* = .021). Linear bending modulus in loading of rib 3 was higher than for ribs 5 and 6 (*p* < .001 for both), and similarly in rib 4 was higher than ribs 5 and 6 (*p* = .001 and *p* < .001, respectively). CVAM was a significant positive covariate (*p* = .001).

Specimen cubic bending moduli in loading (*E*_3*L*_) had a median [range] of 17.0 [1.61-646] GPa (Figure 4). It was larger in females than males (*p* = .019), negatively associated with age (*p* = .016), and positively correlated with amount of calcification (*p* = .001). Cubic bending moduli in loading of ribs 3, 4, and 6 were higher than for rib 5 (*p* = .027, .007, and .048, respectively). CVAM was a significant positive covariate (*p* < .001).

Specimen linear bending moduli in unloading (*E*_1*U*_) had a median [range] of 8.48 [0.47-75.8] MPa (Figure S2). It was larger in females than males (*p* = .047), negatively associated with age (*p* < .001), but was not associated with amount of calcification (*p* = .682). Linear bending modulus in unloading (*E*_1*U*_) of rib 3 was higher than for rib 6 (*p* = .019). CVAM was a not a significant covariate (*p* = .956).

Specimen cubic bending moduli in unloading (*E*_3*U*_) had a median [range] of 16.3 [1.61-646] GPa in the SI direction (Figure S2). It was larger in females than males (*p* = .011), negatively associated with age (*p* = .005), and positively correlated with amount of calcification (*p* = .002). Cubic bending moduli in unloading of ribs 3 and 4 were higher than for rib 5 (*p* = .014 and .004, respectively). CVAM was a significant positive covariate (*p* < .001).

## Discussion

In this study, we investigated the relationships between age, degree of calcification, sex, and rib level on the biomechanics of costal cartilage through whole cartilage characterization. We conducted 4-point bending tests to mechanically characterize whole costal cartilage to better mimic endogenous conditions. By minimizing the degree of preprocessing of the costal cartilage samples (ie, cleaning the samples but leaving the perichondrium), whole cartilage bending facilitated a better representation of the physiologically intact state of the costal cartilage. We found that male sex had larger size and thus larger moments of area of cartilage. As a result, male sex was positively associated with higher bending stiffness compared to female sex, supporting our hypotheses. We also found calcification to be positively associated with bending stiffness and bending moduli as hypothesized. In contrast to our hypothesis, we found that age was not associated with increased calcification. Further, age was negatively associated with bending stiffness and bending moduli.

We found the median [range] for linear bending modulus in loading and unloading was 33.8 [5.12-348] MPa for loading and 8.45 [0.474-75.8] MPa for unloading. Our findings were significantly higher than Alkan et al.^20^ and Grellman et al.,^39^ which found the range of the bending moduli to be 0.39-4.05 and 5.9-11.7 MPa, respectively. This discrepancy is likely due to the removal of perichondrium from the costal cartilage samples evaluated in these studies, whereas the perichondrium in our study was left intact. According to Forman et al.,^2^ the perichondrium makes up 50% of the structural stiffness of costal cartilage. In fact, our findings were similar to Forman et al.^38^ and Gradischar et al.,^9^ in which both studies left the perichondrium on their costal cartilage samples intact and had bending moduli ranges of 4.8-49 and 2.2-60.6 MPa, respectively. The degree of calcification could also have played a role in the discrepancy of bending moduli between our study and comparative studies. Depending on the distribution, location, and microstructure of calcification in costal cartilage, Lau et al.^18^ found that effective moduli ranged from 20 to 66 MPa when calcification volume comprises 18%-24% of costal cartilage volume. The wide range of bending moduli in our study could be in part attributed to the large range in volumetric calcification in our samples (0%-37.6%). It should also be noted that the analytical approach used to analyze the mechanical testing data assumes a homogeneous material and uniform cross-section, thus the modulus values may be higher than for “pure” cartilage. However, estimates of cartilage moduli from uniform beams without calcification can be obtained from the statistical models (by setting calcification percent volume and CVAM to 0) and are presented in Table S3.

Calcification clearly increases stiffness of costal cartilage, as the linear and cubic stiffnesses and elastic moduli in loading were all positively associated with the amount of calcification. Wang et al.^40^ found that calcified costal cartilage samples increased the tensile and compressive Young’s moduli by 30.1% and 126%, respectively. Further, Forman and Kent^28^ found that a volumetric increase of calcification from 0% to 24% could lead to an increase in costal cartilage stiffness of 2.3-3.8 times. Our analysis indicates that increasing calcification from 0% to 24% would increase linear stiffness and modulus in loading by 1.4-1.5 times and cubic stiffness and modulus in loading by 2.1-3.1 times, similar to these prior findings. While prior studies had linked increasing calcification to age,^41^^,^^42^ our study did not find any correlation between degree of calcification and age, which could be a result of our small sample size. The scan resolution was such that “small” calcifications of 1 mm size, as described by Lau et al.,^27^ might be missed due to partial volume effects. This could result in slightly lower estimates of volume of calcification. On the other hand, small diffuse calcifications have a minimal effect on elastic modulus,^18^ so this would probably not contribute notably to the evaluated effect of calcification on material properties. We examined three measurements of costal cartilage calcification, and all displayed similar weak associations with stiffness and modulus in univariate comparisons (Table S2). Indeed, scatterplots highlight the wide variation in outcomes across the ranges of calcification measures (Figures S3 and S4). Interestingly, only volume percentage of calcification remained a contributor in the multivariate analyses, perhaps reflecting that it is the most accurate and precise measure of the amount of calcification among the three.

We found higher age to be negatively correlated with linear bending moduli in loading and unloading. Similarly, Weber et al.^19^ found a negative correlation between age and Young’s moduli of their costal cartilage samples. Guo et al.^43^ found there to be a decrease in tensile strength with increasing age; although, of note, their study consisted of costal cartilage samples from a younger age range (5-25) than that seen in our study. Conversely, Lau et al.^17^ reported no effect of age on the stiffness of the mid-substance of costal cartilage. Although, this finding could be attributed to the use of indentation testing, where the heterogeneous nature of costal cartilage and small testing area could mask the effects of age on the overall structure. While perhaps some uncertainty remains, our results contribute to the growing body of evidence that the material stiffness of costal cartilage decreases with aging.

In relation to sex, linear bending stiffness in loading was found to be larger in males than females, but no differences were found in unloading or cubic stiffness. This is likely due to the larger area moments and geometric properties of male costal cartilage samples compared to female samples in our study. Conversely, linear bending moduli in loading was found to be larger in females than males. This differs from the findings of Guo et al.^43^ and Weber et al.,^19^ which both found no influence of sex on the biomechanical properties of costal cartilage. Despite the lack of studies showing sex-based differences in the biomechanical properties of costal cartilage, forensic scientists have utilized differing patterns of calcification in costal cartilage to identify sex.^30^ It is possible that the differing sex-based calcification patterns could intrinsically affect the biomechanical properties of costal cartilage among males and females. Further research needs to be conducted to further examine the relationship between sex and costal cartilage mechanical properties.

As costal cartilage serves various roles in the thorax, providing structure to the thorax, enabling respiration, and protecting internal structures, different rib levels could have differing biomechanical properties that help facilitate these distinct functions. Yet, there are very few prior studies that have examined potential differences in the biomechanical properties of costal cartilage between rib levels. Our study found the linear bending stiffness in loading of rib 6 was larger than in ribs 3, 4, or 5 about the AP axis and rib 3 was smaller than rib 5 about the AP axis. Similarly, cubic bending stiffness in loading and unloading of rib 6 was larger than in ribs 3, 4, or 5 about both the AP and SI axes, and rib 3 was smaller than rib 5 about both the AP and SI axes. Additionally, the linear bending modulus in loading and unloading of rib 3 was larger than for ribs 5 and 6. Linear bending modulus in unloading for rib 4 was larger than rib 6. Weber et al.^19^ did not find differences between rib levels in unconfined compression testing but were able to find differences via indentation testing. There was a significant difference between the indentation Young’s modulus of ribs 2 and 3, 2 and 6, 2 and 7, and 2 and 8 at 5% strain of indentation testing and between ribs 2 and 3, 2 and 6, and 2 and 7 at 10% strain.^19^

The rib cage performs several functions, including facilitating respiration, providing structural support for the body, and the protection of internal organs. As form follows function, the distinct physiological roles required at varying ribs levels could possibly explain some of the differences in the mechanical properties seen between rib levels in our study. Ribs 1-7 are considered true ribs that directly articulate with the sternum via costal cartilage, which functions like a hinge joint to enable the anterior motion of the sternum during respiration. Ribs 8-10 are false ribs that do not connect to the sternum directly; rather, their costal cartilage junctions merge with the costal cartilage from rib 7. While ribs 8-10 also facilitate respiration via a “bucket-handle” lateral motion, they also serve as a rigid structure to protect internal organs.^11^ The costal cartilage of ribs 6-9 further interfaces with each other via interchondral joints supported by dense fibrous ligaments.^44^ It has been reported that the interchondral joint between the costal cartilage of ribs 6 and 7 limits motion.^45^ The larger bending stiffness in the costal cartilage of rib 6 compared to ribs 3, 4, and 5 could be attributed to the intermediate role of rib 6 participating in respiration as well as its association with the more rigid nature of ribs 8-10 via its connection with rib 7. The smaller bending stiffness of ribs 3, 4, and 5 could be attributed to the need to be flexible and deform to facilitate respiration. It is possible that some elastic moduli differences seen in our study could arise from material adaptation to differences in the cross-sectional size between rib levels and sexes, in which smaller sizes result in higher elastic moduli. However, there is a paucity of literature that examines the effect of size differences on costal cartilage properties, so this remains an area for further study.

Costal cartilage is composed of an inner hyaline cartilage mid-substance with chondrocytes embedded in a matrix of type 2 collagen, surrounded by an outer fibrous connective tissue known as the perichondrium.^46^ Due to the mixed composition and inhomogeneous mid-substance of costal cartilage, a nonlinear model could better characterize its material properties compared to existing linear models. Additionally, by modeling the mechanical behavior of costal cartilage under both loading and unloading forces, the dynamic, global nature of costal cartilage can be better understood. These are especially important considerations when creating computational models of the rib cage to predict automotive collision injuries.^47^ Further, a better understanding of the mechanical properties of costal cartilage would facilitate the production of better biomimetic thoracic implants that are used for reconstruction of the rib cage in cases of trauma and oncologic resection.^48^

An important aspect of our analysis approach is the use of non-linear, pseudo-elastic material models. Many biological soft tissues are known to display non-linear behavior.^33^^,^^34^ While this has been noted in some prior studies of cartilage tissue,^40^^,^^43^ most studies have not evaluated non-linear elastic material properties. In the current study, we include both linear and cubic modulus values. The linear values can be interpreted as the initial stiffness or modulus and may be comparable to some linear results from prior studies. The cubic values represent material stiffening with increased strain. Based on the loading moduli evaluated here, the cartilage stiffens by about 5 times at 5% strain (median 4.9, range 2.1-33), which is a nominal estimate of the upper range of strain experienced by specimens in testing. Thus, nonlinearity is a critical and significant aspect of costal cartilage behavior. The pseudo-elastic evaluation of unloading illustrates the visco-elastic nature of the cartilage material. The linear stiffnesses and moduli for unloading were a fraction of those determined for loading (median 26.5%, range 0.0%-65.3%). This encompasses a similar range as reported for instantaneous vs long-term modulus during stress relaxation tests.^17^^,^^40^ While the constant-rate cyclic testing of this study was not designed to fully evaluate visco-elastic behavior, this result suggests that visco-elastic behavior of the whole costal cartilage in bending was relatively similar to the behavior of costal cartilage material in prior studies using different methods. The non-linear and visco-elastic nature of cartilage material makes comparison between existing studies difficult, as load–displacement results may depend strongly on the ultimate stress or strain achieved, as well as the testing rate.

This study had limitations of note, as well as strengths. There was only limited information regarding the past medical history of each donor, such as cause of death and list of the medications that they were taking at the time of their passing. This is relevant as metabolic disorders and exposure to medications, such as corticosteroids, may play a role in premature calcification of costal cartilage.^49^^,^^50^ Of note, only 4 of the 24 donors were taking corticosteroids. Unfortunately, no information was provided regarding medication dosage or duration, so it would be challenging to extrapolate the potential effects of corticosteroids on the costal cartilage calcification of these donors. Similarly, no information pertaining to chemotherapy or radiotherapy regimens was provided for the donors who had cancer. Thus, it is unknown if these regimens affected costal cartilage properties. The use of four-point bending of whole costal cartilage was designed to mimic more endogenous conditions than some testing methods but is still limited in that physiologic mechanical conditions may not be fully represented by this testing method. The use of fresh, frozen cadaveric tissue further may limit how representative the results are of in vivo cartilage properties, as the process of harvesting, freezing, and thawing the tissue before testing could affect the intrinsic mechanical properties of the tissue. Further, there were five specimens that were fully excluded and nine that had some tests excluded due to damage, testing problems, or data issues. Specimens and data were examined for signs of damage after testing, but it is possible tests from damaged specimens remained in the dataset. We did not aim to test to failure, and our target bending limit of 0.5°/mm length, in combination with specimen sizes, suggests theoretical peak strains during testing in the range 4%-11%. While these calculated values do not indicate that specimens should approach failure, we did not directly measure strains during testing to ensure this. This is a significant limitation, as damage to a specimen could have affected the results in unclear ways. Nonetheless, the final dataset represents successful completion of more than 90% of the planned tests (353/384). It should also be noted that with only 24 donors, but multiple samples within each donor, the power to detect age and sex effects may have been lower than for other fixed effects. However, significant effects were observed, indicating sufficient power in this case. With 91 samples tested from 24 individual donors, half male and half female, ranging in age from 47 to 94 yr, this study includes a larger and more representative sample than most prior studies and is a significant addition to our knowledge of costal cartilage mechanics.

In conclusion, this study uniquely characterized the bending properties of whole costal cartilage specimens and evaluated the effects of age, sex, calcification, and rib level, and shows that all these factors affect these properties. As our study characterizes the mechanical properties of whole costal cartilage with an intact perichondrium, the global properties of the rib cage can be better understood under endogenous conditions and incorporated into computational models of the thorax. The findings of our study can be used to create more representative rib cage models for simulations of injury risk pertaining to age- and disease-related changes, as well as the creation of more biomimetic therapeutic thoracic implants.

## Supplementary Material

Costal_Cartilage_Supplementary_Material_JBMRPlus_ziae153

## Data Availability

The data are available upon reasonable request from the corresponding author.
