# Supplementary material for: Bending properties of human cartilaginous ribs and costal cartilage material vary with age, sex, and calcification
Source: JBMR Plus. 2024 Dec 3;9(1):ziae153. doi: 10.1093/jbmrpl/ziae153 (PMC11653008; doi:10.1093/jbmrpl/ziae153)
Supplement: Costal_Cartilage_Supplementary_Material_JBMRPlus_ziae153 [file costal_cartilage_supplementary_material_jbmrplus_ziae153.docx]

Supplemental Table 1: Fourth Moments of Area (mean ± STD)

| Sex | Rib | Area Moment - AP (*I_4AP_*, mm^4^) | Area Moment - SI (*I_4SI_*, mm^4^) |
| --- | --- | --- | --- |
| Male | 3 | 0.117 ± 0.0596 | 0.0150 ± 0.00975 |
|  | 4 | 0.125 ± 0.0609 | 0.0209 ± 0.0133 |
|  | 5 | 0.112 ± 0.0572 | 0.0358 ± 0.0190 |
|  | 6 | 0.113 ± 0.0762 | 0.0763 ± 0.0377 |
| Female | 3 | 0.0425 ± 0.0287 | 0.00454 ± 0.00416 |
|  | 4 | 0.0399 ± 0.0206 | 0.00612 ± 0.00323 |
|  | 5 | 0.0346 ± 0.0203 | 0.0117 ± 0.0101 |
|  | 6 | 0.0345 ± 0.0177 | 0.0241 ± 0.0194 |

Supplemental Table 2: r^2^ (p-values) for univariate associations of Stiffness and Modulus with three measures of costal cartilage calcification. See Supplemental Plots x to y for a view of the associated data. Classifications are None, Central, Peripheral, and Mixed.

| Stiffness | K_1L_ | K_3L_ | K_1U_ | K_3U_ |
| --- | --- | --- | --- | --- |
| % Length | 0.05 (<0.001) | 0.08 (<0.001) | 0.02 (0.013) | 0.10 (<0.001 |
| % Volume | 0.00 (0.934) | 0.02 (0.005) | 0.02 (0.008) | 0.02 (0.003) |
| Classification | 0.14 (<0.001) | 0.07 (<0.001) | 0.02 (0.401) | 0.09 (<0.001) |
| Modulus | E_1L_ | E_3L_ | E_1U_ | E_3U_ |
| % Length | 0.02 (0.004) | 0.03 (0.002) | 0.00 (0.800) | 0.03 (0.002) |
| % Volume | 0.04 (<0.001) | 0.06 (<0.001) | 0.01 (0.036) | 0.06 (<0.001) |
| Classification | 0.02 (0.383) | 0.03 (0.041) | 0.02 (0.419) | 0.03 (0.052) |

Supplemental Table 3: Point estimates [95% CI] of linear and cubic modulus in loading and unloading for pure, uniform cartilage by sex and rib level. Values are estimated from statistical models assuming no calcification, no variation in cross-section along the specimen length, and Age of 70.8 years (average in the donors).

| Rib | E_1L_  (MPa) | E_3L_  (GPa) | E_1U_  (MPa) | E_3U_  (GPa) |
| --- | --- | --- | --- | --- |
| Male |  |  |  |  |
| 3 | 26.8 [21.6 - 33.3] | 10.8 [7.5 - 15.5] | 8.0 [4.2 - 15.3] | 10.8 [7.6 - 15.4] |
| 4 | 24.4 [19.5 - 30.6] | 11.4 [7.8 - 16.6] | 7.1 [3.5 - 14.5] | 11.3 [7.8 - 16.5] |
| 5 | 19.6 [15.5 - 24.7] | 8.4 [5.7 - 12.4] | 5.8 [2.7 - 12.4] | 8.2 [5.6 - 12.0] |
| 6 | 18.4 [14.6 - 23.2] | 10.5 [7.1 - 15.5] | 4.0 [1.9 - 8.4] | 9.7 [6.6 - 14.2] |
| Female |  |  |  |  |
| 3 | 45.6 [35.9 - 58.1] | 18.0 [12.0 - 27.1] | 15.0 [7.0 - 31.9] | 18.5 [12.4 - 27.6] |
| 4 | 41.5 [32.2 - 53.4] | 19.0 [12.3 - 29.2] | 13.3 [5.9 - 30.1] | 19.4 [12.7 - 29.6] |
| 5 | 33.3 [25.8 - 42.9] | 14.0 [9.1 - 21.6] | 10.9 [4.8 - 24.6] | 14.1 [9.2 - 21.4] |
| 6 | 31.2 [24.2 - 40.3] | 17.5 [11.4 - 26.9] | 7.4 [3.3 - 16.8] | 16.7 [11.0 - 25.4] |


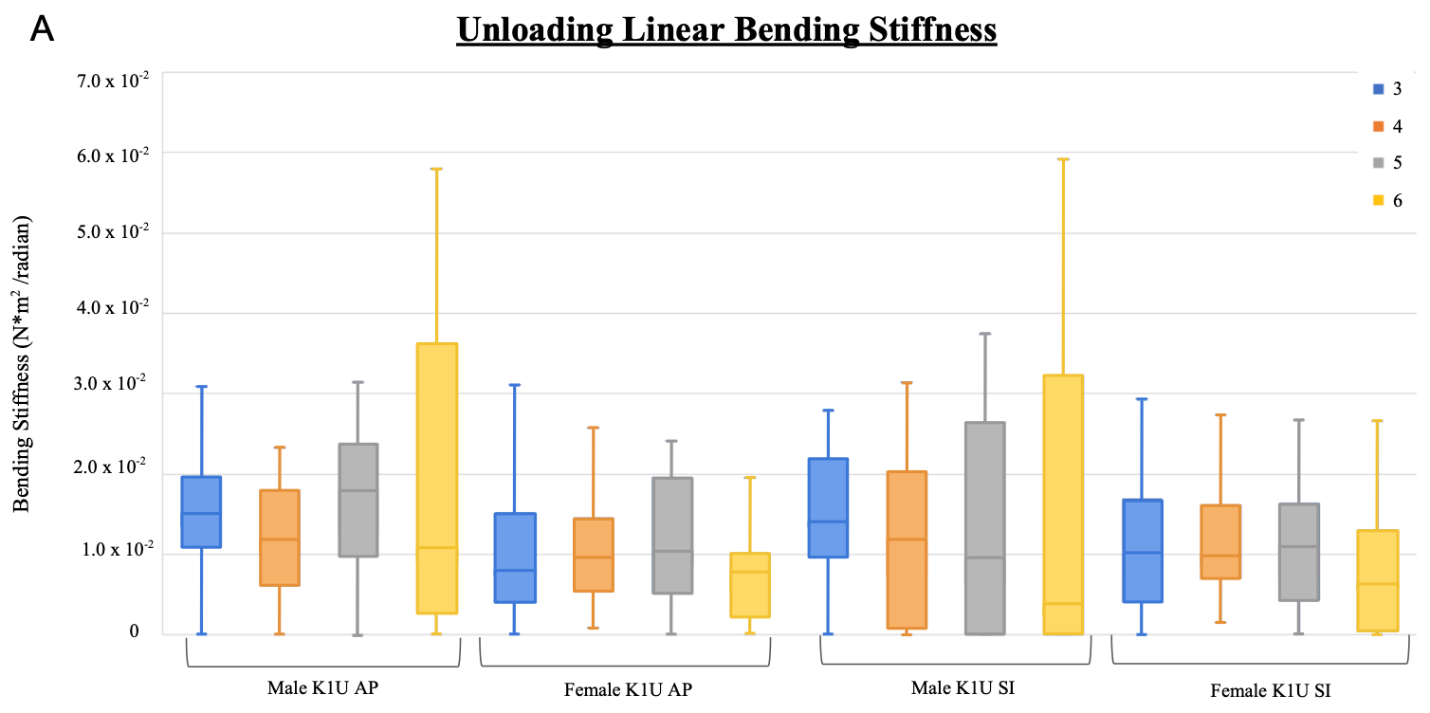

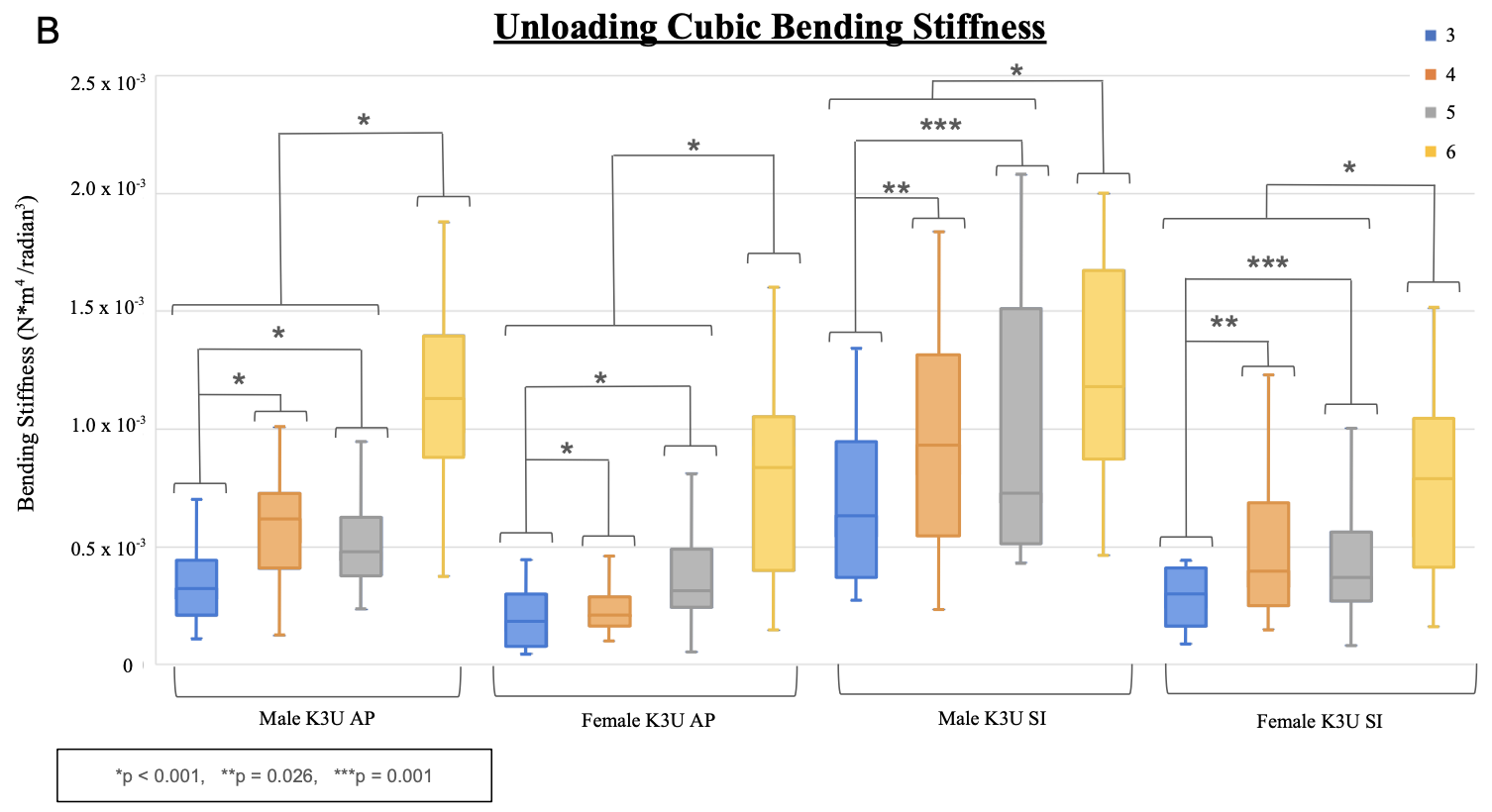
Supplemental Figure 1: A) Linear unloading (K1U) bending stiffness and B) cubic unloading (K3U) bending stiffness averaged across rib levels along anterior-posterior (AP) and superior-inferior (SI) axes.  Whiskers show range, excluding outliers > 1.5IQR outside the 1^st^ and 3^rd^ quartiles.


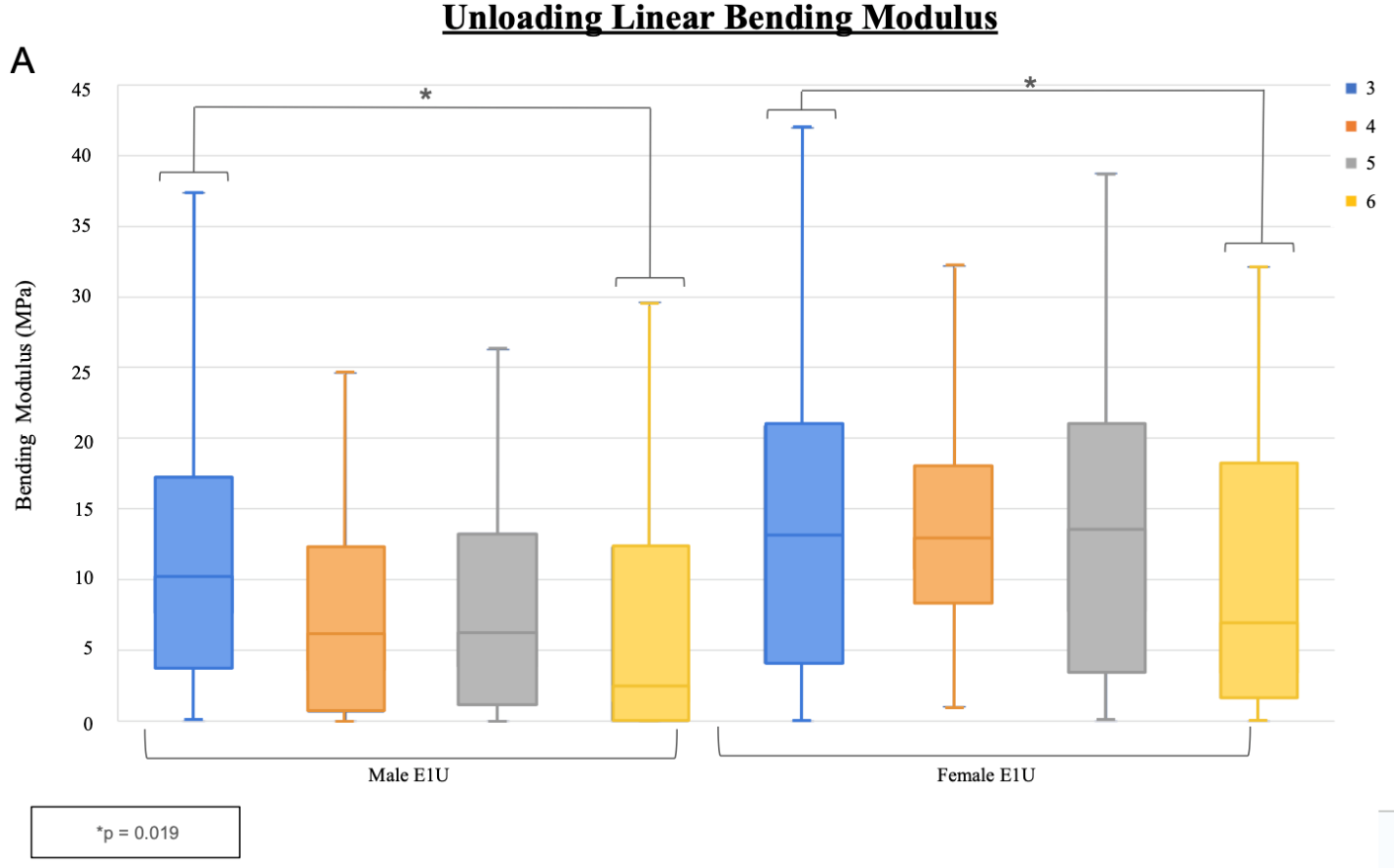

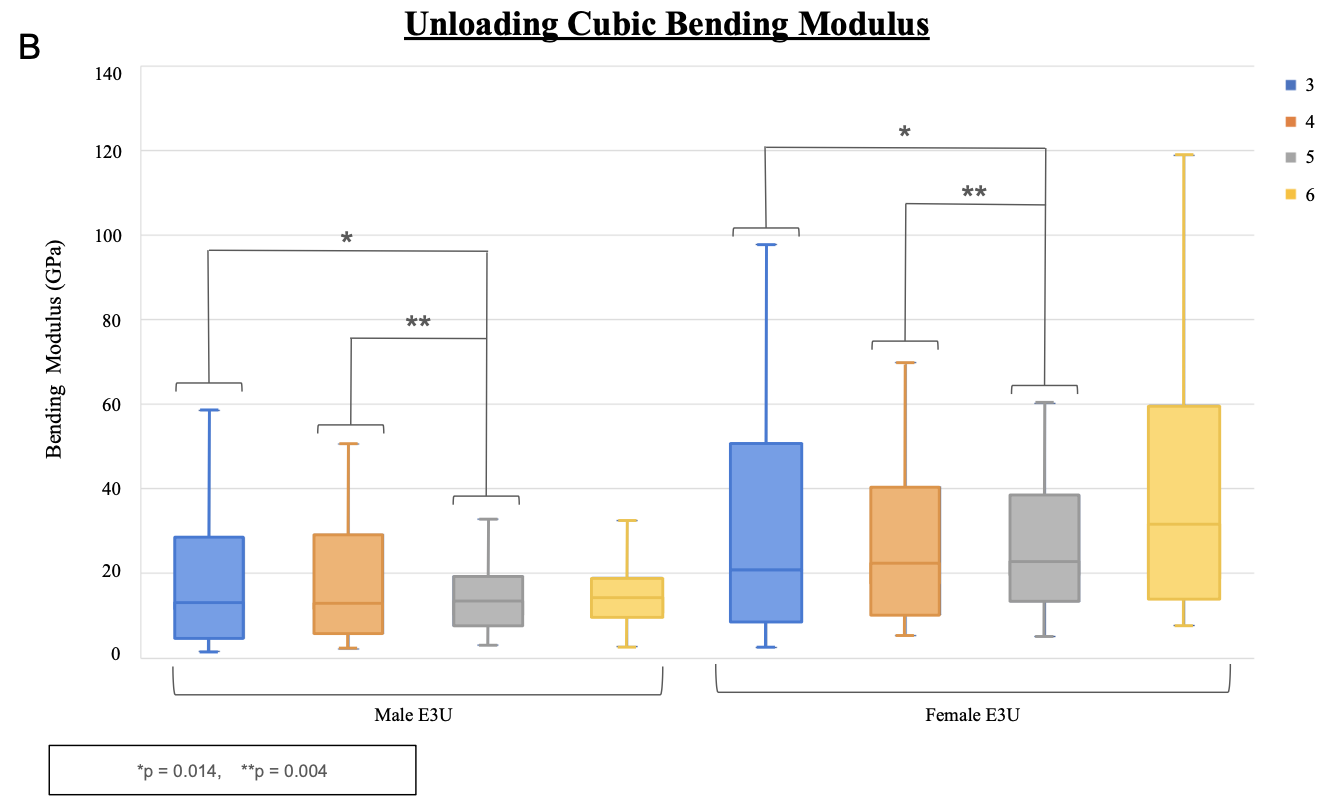
Supplemental Figure 2: A) Linear unloading (E1U) bending elastic modulus and B) cubic unloading (E3U) bending modulus by rib level and sex. Whiskers show range, excluding outliers > 1.5IQR outside the 1^st^ and 3^rd^ quartiles.

| A 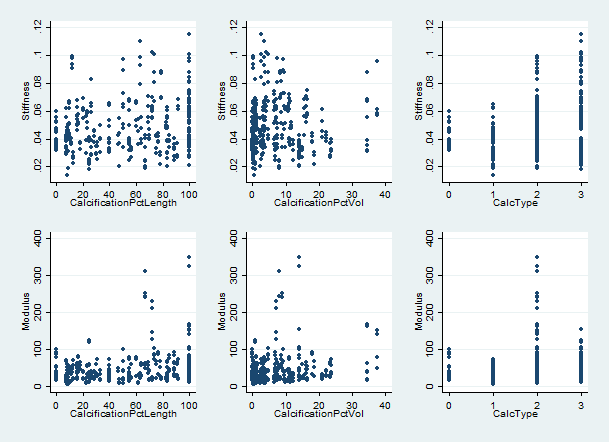 |
| --- |
| B 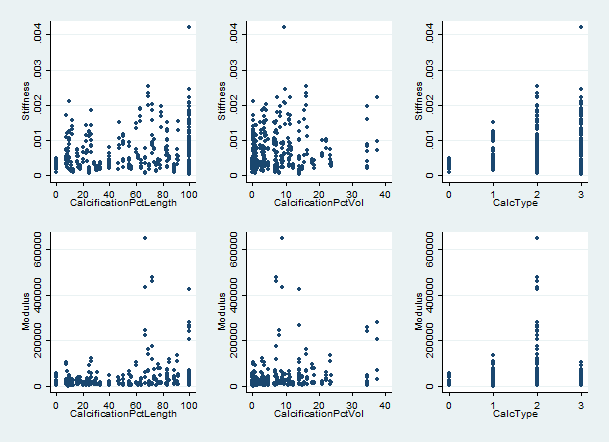 |

Supplemental Figure 3: Scatterplots of Stiffness (N-m^2^/rad) and Modulus (MPa) for Loading versus measures of costal cartilage calcification. A) Linear stiffness and modulus (K1L, E1L). B) Nonlinear stiffness and modulus (K3L, E3L). Calcification type = 0: None; 1: Central, 2: Mixed; 3: Peripheral.

| A 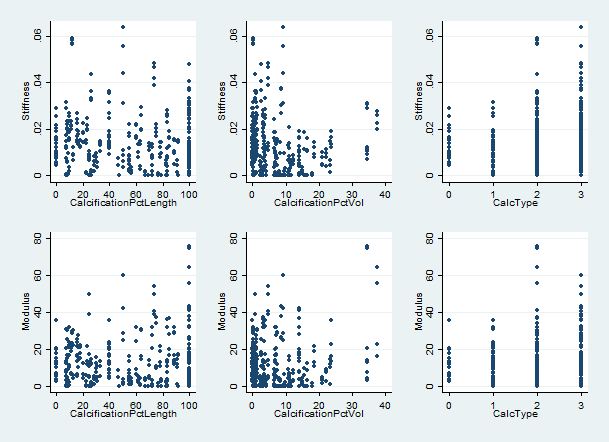 |
| --- |
| B 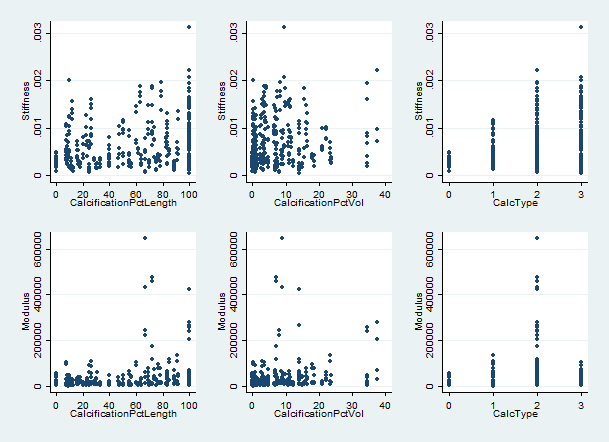 |

Supplemental Figure 4: Scatterplots of Stiffness (N-m^4^/rad) and Modulus (MPa) for Unloading versus measures of costal cartilage calcification. A) Linear stiffness and modulus (K1U, E1U). B) Nonlinear stiffness and modulus (K3L, E3L). Calcification type = 0: None; 1: Central, 2: Mixed; 3: Peripheral.
